# Inflammation associated ethanolamine facilitates infection by Crohn's disease-linked adherent-invasive *Escherichia coli*

**DOI:** 10.1016/j.ebiom.2019.03.071

**Published:** 2019-04-26

**Authors:** Michael J. Ormsby, Michael Logan, Síle A. Johnson, Anne McIntosh, Ghaith Fallata, Rodanthi Papadopoulou, Eleftheria Papachristou, Georgina L. Hold, Richard Hansen, Umer Z. Ijaz, Richard K. Russell, Konstantinos Gerasimidis, Daniel M. Wall

**Affiliations:** a Institute of Infection, Immunity and Inflammation, College of Medical, Veterinary and Life Sciences, Sir Graeme Davies Building, University of Glasgow, Glasgow G12 8TA, United Kingdom; b School of Engineering, University of Glasgow, Glasgow, Rankine Building, 79-85 Oakfield Ave, Glasgow G12 8LT, United Kingdom; c Human Nutrition, School of Medicine, College of Medical Veterinary and Life Sciences, University of Glasgow, Glasgow Royal Infirmary, Glasgow G31 2ER, United Kingdom; d Microbiome Research Centre, St George and Sutherland Clinical School, UNSW, Australia; e Department of Pediatric Gastroenterology, Hepatology and Nutrition, Royal Hospital for Children, 1345 Govan Road, Glasgow G51 4TF, United Kingdom

**Keywords:** Ethanolamine, Adherent-invasive *Escherichia coli*, Biomarker, Crohn's disease

## Abstract

**Background:**

The predominance of specific bacteria such as adherent-invasive *Escherichia coli* (AIEC) within the Crohn's disease (CD) intestine remains poorly understood with little evidence uncovered to support a selective pressure underlying their presence. Intestinal ethanolamine is however readily accessible during periods of intestinal inflammation, and enables pathogens to outcompete the host microbiota under such circumstances.

**Methods:**

Quantitative RT-PCR (qRT-PCR) to determine expression of genes central to ethanolamine metabolism; transmission electron microscopy to detect presence of bacterial microcompartments (MCPs); *in vitro* infections of both murine and human macrophage cell lines examining intracellular replication of the AIEC-type strain LF82 and clinical *E. coli* isolates in the presence of ethanolamine; determination of *E. coli* ethanolamine utilization (*eut*) operon transcription in faecal samples from healthy patients, patients with active CD and the same patients in remission following treatment.

**Results:**

Growth on the intestinal short chain fatty acid propionic acid (PA) stimulates significantly increased transcription of the *eut* operon (fold change relative to glucose: >16.9; *p*-value <.01). Additionally ethanolamine was accessible to intra-macrophage AIEC and stimulated significant increases in growth intracellularly when it was added extracellularly at concentrations comparable to those in the human intestine. Finally, qRT-PCR indicated that expression of the *E. coli eut* operon was increased in children with active CD compared to healthy controls (fold change increase: >4.72; *P* < .02). After clinical remission post-exclusive enteral nutrition treatment, the same CD patients exhibited significantly reduced *eut* expression (Pre *vs* Post fold change decrease: >15.64; *P* < .01).

**Interpretation:**

Our data indicates a role for ethanolamine metabolism in selecting for AIEC that are consistently overrepresented in the CD intestine. The increased *E. coli* metabolism of ethanolamine seen in the intestine during active CD, and its decrease during remission, indicates ethanolamine use may be a key factor in shaping the intestinal microbiome in CD patients, particularly during times of inflammation.

**Fund:**

This work was funded by Biotechnology and Biological Sciences Research Council (BBSRC) grants BB/K008005/1 & BB/P003281/1 to DMW; by a Tenovus Scotland grant to MJO; by Glasgow Children's Hospital Charity, Nestle Health Sciences, Engineering and Physical Sciences Research Council (EPSRC) and Catherine McEwan Foundation grants awarded to KG; and by a Natural Environment Research Council (NERC) fellowship (NE/L011956/1) to UZI. The IBD team at the Royal Hospital for Children, Glasgow are supported by the Catherine McEwan Foundation and Yorkhill IBD fund. RKR and RH are supported by NHS Research Scotland Senior fellowship awards.

**Research in context**

**Evidence before this study**

Adherent-invasive *Escherichia coli* (AIEC) have been implicated in the aetiology of Crohn's disease (CD), being isolated in consistently greater numbers from CD patients compared to healthy controls. The reasons underlying this association however are poorly understood. Additionally the ability of AIEC to replicate to high numbers within macrophages indicated that a readily available carbon source must be present in the intestine.

**Added value of this study**

In this study we have determined that the intestinal short chain fatty acid propionic acid acts as a signal for AIEC to alter their metabolism and increase their use of ethanolamine, an intestinal metabolite known to be used by pathogens during times of inflammation. To date the rapid replication of AIEC in macrophages has been unexplained, but we have shown here that this rapid intracellular growth can be facilitated by the presence of extracellular levels of ethanolamine comparable to those in the human intestine. Lastly, we have shown the clinical relevance of our findings by detailing the increased metabolism of ethanolamine by *E. coli* in pediatric patients with active CD, and a significant reduction upon remission in the same patients.

**Implications of all the available evidence**

Our study has revealed an important role for PA as a signaling molecule for AIEC, allowing it to adapt to life in the inflamed CD intestine through the use of ethanolamine. The ability to utilize ethanolamine, which is released during times of intestinal inflammation, renders AIEC able to out-compete commensal microbes under the conditions seen in CD. The increased *E. coli* metabolism of ethanolamine seen in pediatric CD patients with active disease, when compared to healthy controls and those in remission, strongly suggests that ethanolamine is a key metabolite in the shaping CD microbiome.

Alt-text: Unlabelled Box

## 1 Introduction

Adherent-invasive *E. coli* (AIEC) are over-represented in the ileal microbiota of Crohn's disease (CD) patients, being present in 51.9% of mucosal samples from CD patients compared with 16.7% in healthy controls [[1], [2], [3], [4], [5]]. Alterations in the gut microbiota composition of patients suffering from CD are well reported with the majority of studies reporting an increase in the abundance of Proteobacteria, of which AIEC are members, and a decrease in Firmicutes [6,7]. While AIEC strains harbor genetic similarity to extra-intestinal pathogenic *E. coli* (ExPEC) in terms of phylogenetic origin and virulence genotype, the factors underlying their virulence have proved more difficult to identify [8]. In addition, the discovery of AIEC strains across all five major diverse phylogroups of *E. coli* means that an overarching explanation for the origin and virulence of AIEC has remained out of reach.

Intestinal pathogens utilize various mechanisms to outcompete the host intestinal microbiota, thus increasing their ability to persist and cause disease. These mechanisms include induction of inflammation, direct or indirect killing of commensals, or exploitation of alternative carbon sources [[9], [10], [11]]. Intestinal pathogens use a variety of carbon sources during infection: *Escherichia coli* and *Clostridium perfringens* using sialic acid [12,13]; Enterohaemorraghic *E. coli* (EHEC) consume galactose, hexuronates and ribose [14]; while *Yersinia enterocolita* and *Salmonella enterica* serovar Typhimurium use the adenosyl-cobalamin, 1,2-Propanediol degradation (1,2-PD; *pdu*) and tetrathionate operons in concert to catabolize 1,2-PD under the anaerobic conditions found in the gut [15]. Recent evidence also suggested a role for 1,2-PD metabolism during adherent-invasive *E. coli* (AIEC) colonization with the *pdu* operon shown to be overrepresented within this CD-associated pathotype and possibly playing a role in driving systemic inflammation, although other work has questioned this link [[16], [17], [18]].

Along with 1,2-PD, phosphatidylethanolamine, a ubiquitous component of host cell membranes, is abundant in the inflamed intestine and is readily hydrolysed into ethanolamine and glycerol [19,20]. Ethanolamine can be used as a carbon and nitrogen source by a variety of intestinal pathogens such as *S.* Typhimurium, enterohaemorrhagic *E. coli* (EHEC), *Enterococcus faecalis*, *Listeria monocytogenes* and *Clostridium difficile* [[21], [22], [23], [24], [25]]. Inflammation associated with infection renders 1,2-PD and ethanolamine available for metabolism as reduced tetrathionate is released allowing its use as a terminal electron acceptor for growth on these carbon sources. The inflammatory environment of the CD intestine similarly offers access to these alternative carbon sources as tetrathionate is again released and available to facilitate 1,2-PD and ethanolamine metabolism [21,23,26,27]. Although critical to outgrowth of intestinal pathogens during inflammation, many bacteria cannot readily use these carbon sources [17,22,28].

Recently, we have shown that exposure of AIEC to propionic acid (PA), an abundant intestinal short chain fatty acid (SCFA), results in modulation of the key phenotypic traits of the AIEC pathotype, rendering PA-exposed bacteria more adherent, invasive and persistent [29]. This is in stark contrast to the antimicrobial and anti-virulence effects PA exerts on other intestinal pathogens such as *S*. Typhimurium and *Campylobacter spp*. [[30], [31], [32], [33], [34], [35], [36], [37], [38]]. Here we show that the intestinal SCFA PA causes AIEC to significantly increase its ethanolamine metabolism. To overcome the toxic by-products associated with ethanolamine use, AIEC synthesize and then excrete bacterial microcompartments (MCPs). Additionally, ethanolamine added extracellularly to macrophages, at concentrations comparable to those of the human intestine, stimulated rapid intracellular proliferation of AIEC. Finally, we determined the clinical relevance of these findings, establishing that despite *E. coli* numbers remaining unchanged in patients with active CD, ethanolamine use was significantly increased. However, ethanolamine metabolism was significantly reduced in these same patients upon treatment leading to clinical remission.

## 2 Methods

### 2.1 Bacterial strains and growth conditions

Strains used in this study are listed in Supplementary Table S1 and were routinely grown at 37 °C at 180 rpm in Lysogeny broth (LB) or M9 minimal medium ([20% M9 salts (32 g Na_2_H_2_PO_4_2H_2_O, 12.5 g NaCl, 2.5 g NH_4_Cl, 7.5 g KH_2_PO_4_ and 400 ml H_2_O], 0.1% trace metal solution, 0.2 mM MgSO_4_, 0.02 mM CaCl_2_, 1 mM thiamine, 0.01% 5 g/l FeCl_3_, 0.01% 6.5 g/l ethylenediaminetetraacetic acid (EDTA), 0.1% taurocholic acid and dH_2_O) supplemented with d-glucose (10 mM), sodium propionate (PA; 20 mM), 1,2-propanediol (1,2-PD; 20 mM) or ethanolamine (ethanolamine; 20 mM). Strains for infection were grown overnight in 10 ml cultures of RPMI-1640 supplemented with 3% fetal calf serum (FCS; heat-inactivated) and 2 mM L-glutamine before being back-diluted the following morning into 10 ml of the same media. These were then grown at 37 °C at 180 rpm to an optical density at 600 nm (OD_600_) of 0.6 before further dilution to give a final multiplicity of infection (MOI) of 10. For transmission electron microscopy (TEM), isolates were grown in No-Carbon-E (NCE) media supplemented with 20 mM glucose or PA at 37 °C, to an OD_600_ of 0.6. For real-time PCR (qRT-PCR), bacteria were grown aerobically in NCE media [39]. Twenty millimolar PA, 1,2-PD, ethanolamine or d-glucose were added with 200 nM cyano-cobalamin to act as an electron acceptor [40]. Cultures were grown overnight in LB, washed three times in NCE media with no carbon source added, and inoculated 1:100 into 10 ml NCE media containing each respective carbon source. Cultures were grown until mid-log phase (OD_600_ of 0.6) and used for RNA-extraction. *eutR* deletion strains were generated by Lambda red-mediated mutagenesis as previously described [41] using the primers *eutR* KO For and eutR KO Rev (Supplementary Table S2). *eutR* deficient strains were confirmed using the primers Δ*eutR* Check For and Δ*eutR* Check Rev All chemical suppliers are listed in Supplementary Table S4.

Clinical isolates (B94, B115, B122 and B125) were from the “Bacteria in Inflammatory bowel disease in Scottish Children Undergoing Investigation before Treatment” (BISCUIT) study [42]. All isolates were recovered from patients with Crohn's disease. The median (range) age was 13.7 (11.2 to 15.2), height z-score was −0.4 (−2.0 to 0.2), weight z-score was −0.7 (−3.4 to −0.1), and BMI z-score was −1.3 (−4.0 to 0.4). Symptom duration prior to diagnosis was median 7.5 months [[5], [6], [7], [8], [9], [10], [11], [12]]. 50% had granulomas present on initial histology. Phenotypes by Paris criteria [43] at diagnosis were: B94- colonic, non-stricturing/non-penetrating (L2, B1); B115- colonic, non-stricturing/non-penetrating (L2, B1); B122- ileocolonic, stricturing (L3, B2); B125- ileocolonic, non-stricturing/non-penetrating (L3, B1). This study is publically registered on the United Kingdom Clinical Research Network Portfolio (9633).

### 2.2 Transmission electron microscopy

Five microlitre suspension droplets were placed onto the glow discharged surface of 300 mesh Formvar/carbon coated nickel grids and left to settle for 2 min. Grids were then placed sample side down onto a 30 μl droplet of 2% ammonium molybdate for 30 s prior to air-drying. Samples were viewed on a FEI Tecnai T20 TEM running at 200 kV and images captured using a GATAN Multiscan 794 camera and GATAN Digital Imaging software (DM4 converted to TIFF).

### 2.3 Total RNA extraction

Bacterial cultures were grown as above and mixed with two volumes of RNAprotect reagent, before incubating for 5 min at room temperature. Total RNA was extracted and genomic DNA removed as described previously [44].

### 2.4 Patient faecal samples

The whole bowel movement was collected, stored in a cool bag under anaerobic conditions (Oxoid™ AnaeroGen™) and transferred to the laboratory within three hours of defecation [45]. The whole sample was homogenized with mechanical kneading and aliquots were stored in RNAlater at −70 °C.

Samples were collected from 10 newly diagnosed, treatment naïve children (11.4 (Q1:8.5, Q3:15.3 years; 5 female) with active CD undergoing an 8-week induction treatment with exclusive enteral nutrition (EEN) with a polymeric casein-based liquid feed (Modulen) as described previously [46]. No other food was allowed. A first sample was collected before EEN initiation and another at the end of the eight-week treatment. A single faecal sample was collected from healthy children with no family history of inflammatory bowel disease to serve as a control group. Children with CD and healthy controls were matched for age and gender. Participants that had received antibiotics three months before or during the study were excluded.

Disease activity was monitored during the course of the EEN treatment using the weighted pediatric CD Activity Index (wPCDAI) [47]. Faecal calprotectin (FC), an established marker of colonic inflammation was measured with the Calpro ELISA kit as described previously (Supplementary Table S3) [48].

#### 2.4.1 Ethics statement

Patients were recruited from the pediatric gastroenterology clinics at the Royal Hospital for Children, Glasgow and healthy controls from the same background community using leaflet advertisement. Children with CD were diagnosed according to the revised Porto criteria [49]. All participants and their carers signed informed consent. The study was approved by the NHS West of Scotland Research Ethics Committee (14/WS/1004) and was registered at www.clinicaltrials.gov (NCT02341248).

### 2.5 Total RNA extraction from faecal samples

Faecal samples were stored at −80 °C in RNAlater. To extract RNA, samples were thawed on ice before brief centrifugation to remove RNAlater. Approximately 250 μg of faecal material was then subjected to RNA isolation using the RNeasy PowerMicrobiome Kit. RNA quantity and quality was estimated using a NanoDrop (ThermoFisher Scientific) spectrophotometer and DNA depletion confirmed through PCR using 16S primers (Supplementary Table S2).

### 2.6 Quantitative real-time PCR (qRT-PCR)

cDNA was generated from total RNA using an Affinity Script cDNA multi-temp Synthesis Kit following the manufacturer's instructions. Levels of transcription were analysed by qRT-PCR using PerfeCTa SYBR Green FastMix. Individual reactions were performed in triplicate within each of three biological replicates. The 16S rRNA gene was used to normalize the results. RT-PCR reactions were carried out using the CFX Connect Real-Time PCR Detection System (BIO-RAD Laboratories, Inc.) according to the manufacturer's specifications and the data were analysed according to the 2^-ΔΔCT^ method [50]. All primers used are listed in Supplementary Table S2.

### 2.7 Cell culture and maintenance

The RAW264.7 murine macrophage cell line obtained from the American Type Culture Collection (ATCC) was maintained in RPMI-1640 medium supplemented with 10% FCS, 2 mM l-glutamine and penicillin/streptomycin. THP-1 (ECACC 88081201) cells were obtained from the European Collection of Authenticated Cell Cultures (ECACC) as growing cultures. Cells were maintained in 10% FCS, 2 mM l-glutamine and penicillin/streptomycin. All cells were maintained at 37 °C and 5% CO_2_ with regular media changes.

### 2.8 Cell culture infection

Both RAW264.7 macrophages and THP-1 cells were seeded at 1 × 10^5^ cells per well of a 24-well plate 48 h prior to infection. THP-1 cells were differentiated for 24 h in the presence of 200 nM phorbol 12-myristate 13-acetate (PMA) to activate macrophages. After activation, the medium was removed, and the cells washed prior to infection to remove dead or non-adherent cells and left for a further 24 h. RAW264.7 macrophages were treated with 100 ng/ml lipopolysaccharide to induce an activated state 24 h after seeding. Infections for both cell types were carried out in RPMI media supplemented with 3% FCS and L-glutamate. Infections were carried out at an MOI of 10. After 1 h the bacteria that had not been internalized were killed by adding 50 µg/ml gentamycin sulfate (Sigma-Aldrich) and the infection allowed to proceed.

### 2.9 Statistical analysis

All statistical tests were performed with GraphPad Prism software. All replicates in this study were biological; that is, repeat experiments were performed with freshly grown bacterial cultures or mammalian cell lines, as appropriate. Technical replicates of individual biological replicates were also conducted, and averaged. Values are represented as means ± standard deviation. Significance was determined using *t*-tests (multiple and individual as indicated in the figure legends) and ANOVA (one-way or two-way) corrected for multiple comparisons with a Tukey's *post hoc* test (as indicated in the figure legends). qRT-PCR data was log-transformed before statistical analysis. For patient samples statistical analyses were preformed using GraphPad Prism, with data analysed by one-way ANOVA followed by Dunns multiple comparisons post-test. Values were considered statistically significant when *P* values were *P < .05, **P < .01, ***P < .001.

## 3 Results

### 3.1 The intestinal short chain fatty acid PA stimulates AIEC degradation of ethanolamine

**Fig. 1:**
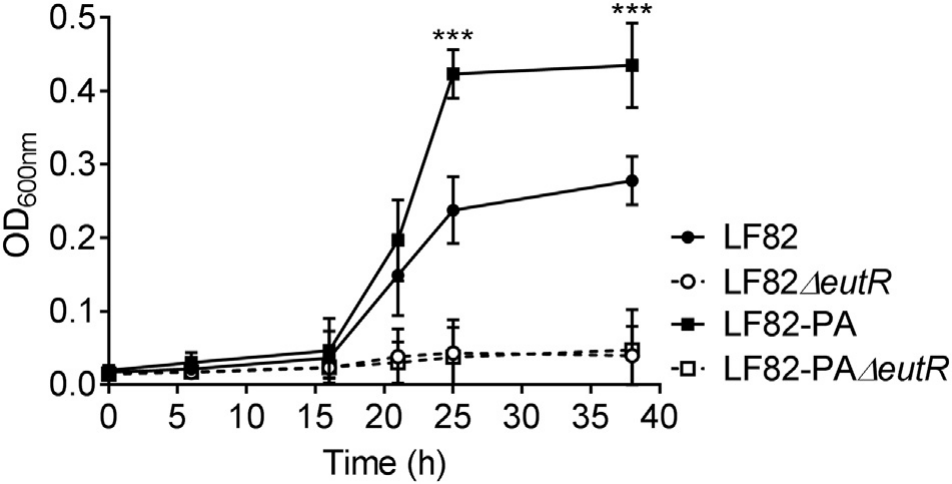
PA stimulates AIEC degradation of ethanolamine. Anaerobic growth (OD_600nm_) of LF82, LF82-PA and their corresponding *eutR* knock out mutants in minimal media (NCE) supplemented with ethanolamine (20 mM), MgSO_4_ (1 mM), trace metals and sodium thiosulphate (40 mM). Data were analysed using a two-way ANOVA with Tukey; *p* < .001 ***. Only significant differences between LF82 and LF82-PA are shown. There were no significant differences between mutant strains at any time point.

**Supplementary Fig. S1:**
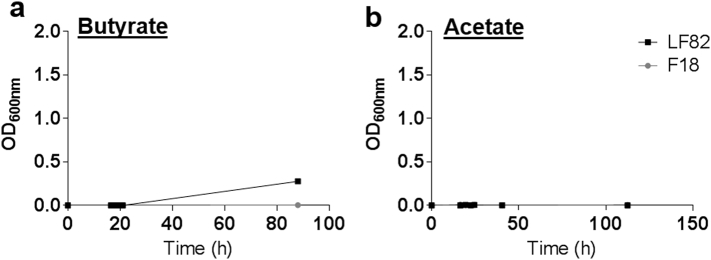
AIEC are unable to utilize butyrate or acetate as sole carbon sources for effective growth. The growth of commensal (F-18) and adherent and invasive (LF82) *E. coli* strains in minimal media (M9) supplemented with 20 mM butyrate (a) or acetate (b) was monitored over time.

We previously observed that pre-exposure of the wild type AIEC strain LF82 to PA (termed LF82-PA), resulted in a more virulent phenotype [29]. Comparing growth of LF82-PA on ethanolamine as a sole carbon source relative to wild type LF82, indicated that LF82-PA could utilize ethanolamine more rapidly resulting in higher biomass (doubling time [OD_600nm_ 0.1 to 0.2] LF82-PA: 2.5 h; LF82: 6.7 h) (Fig. 1). No difference between the LF82 and LF82-PA strains was noted previously in rich nutrient media, indicating this was not a universal increase in growth rate post-PA exposure [29]. Subsequent deletion of *eutR*, the regulator of the *eut* operon, removed the ability of LF82 and LF82-PA to grow on ethanolamine (Fig. 1). Despite long-term exposure, LF82 was unable to efficiently metabolise the other predominant intestinal SCFAs, acetate and butyrate, as sole carbon sources (Supplementary Fig. S1).

### 3.2 Ethanolamine degradation occurs in bacterial microcompartments (MCPs)

**Fig. 2:**
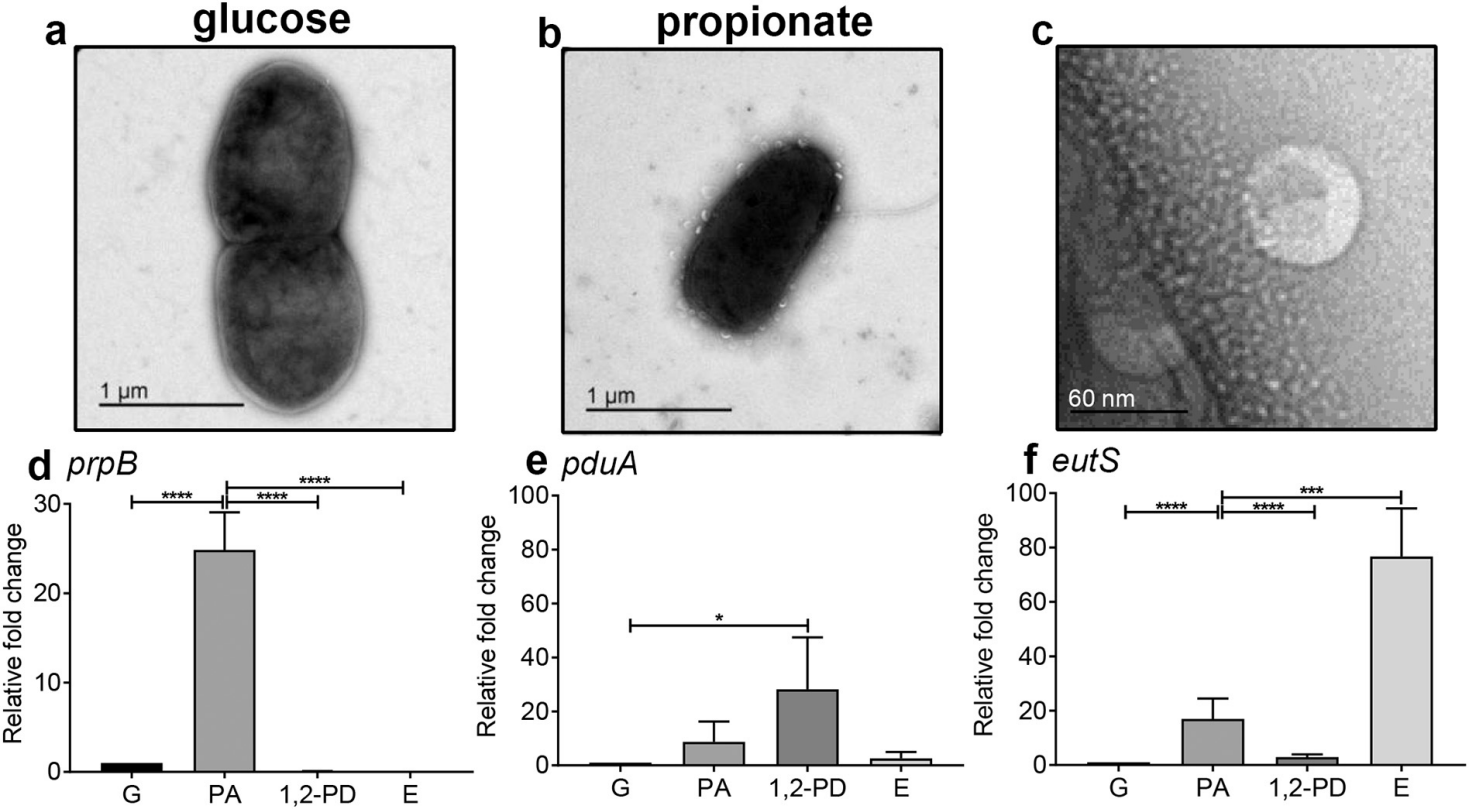
Growth on PA stimulates the production of bacterial MCPs for the utilization of ethanolamine as a carbon source. For TEM, cultures of LF82 were grown in NCE media supplemented with cobalamin (200 nM) and either (a) glucose or (b) PA, at a final concentration of 20 mM. (c) A close up of a MCP-containing outer membrane vesicle from a PA supplemented culture is shown. qRT-PCR was conducted on LF82 grown in NCE media supplemented with cobalamin (200 nM) and either glucose (G), propionic acid (PA) 1,2-propanediol (1,2-PD) or ethanolamine (E) at a final concentration of 20 mM. Relative fold change of (d) *prpB*, (e) *pduC* and (f) *eutS* were measured relative to their expression in the presence of glucose, using 16S rRNA as an internal control. Four independent biological replicates were performed. Data are expressed as relative fold change ± SD and were analysed using a one-way ANOVA with Tukey; *p* < .05 *; p < .001 ***.

In the presence of the intestinal SCFA PA, transmission electron microscopy (TEM) revealed the release of outer membrane vesicles containing pentagonal shapes that we speculated to be MCP containing vesicles (Fig. 2a). MCPs are utilized by bacteria for growth on 1,2-PD and ethanolamine as their metabolism releases the toxic by-products propanol and ethanol that can then be sequestered into the MCP to protect the bacteria. We first examined the ability of LF82 to express MCPs from either the *eut* or *pdu* operons, which are known to encode MCPs. Outer membrane vesicles were not detected during growth on glucose (Fig. 2a).

To further understand the role of MCPs in ethanolamine metabolism and their link to intestinal SCFA levels, qRT-PCR examination of the genes central to PA metabolism (*prpB*) and MCP production (*eutS* and *pduC*) was undertaken in the presence of the relevant carbon sources. This revealed that, as expected, transcription of *prpB* was highest during growth on PA (fold change relative to glucose: 24.8; *p*-value <.0001); *pduC* was highest during growth on 1,2-PD (fold change relative to glucose: 28.2; p-value <.05); and *eutS* was highest during growth on ethanolamine (fold change relative to glucose: 76.7; p-value <.001). However, growth on the intestinal SCFA PA stimulated significantly increased transcription of the *eut* operon (fold change relative to glucose: >16.9; p-value <.01) while the *pdu* operon was not significantly affected by PA presence (fold change relative to glucose: >8.4; p-value 0.79). Therefore exposure of LF82 to PA induces significant metabolic changes that adapt bacteria to utilize a carbon and nitrogen source readily available in the inflamed intestine.

### 3.3 Extracellular ethanolamine stimulates increased AIEC intra-macrophage replication

**Fig. 3:**
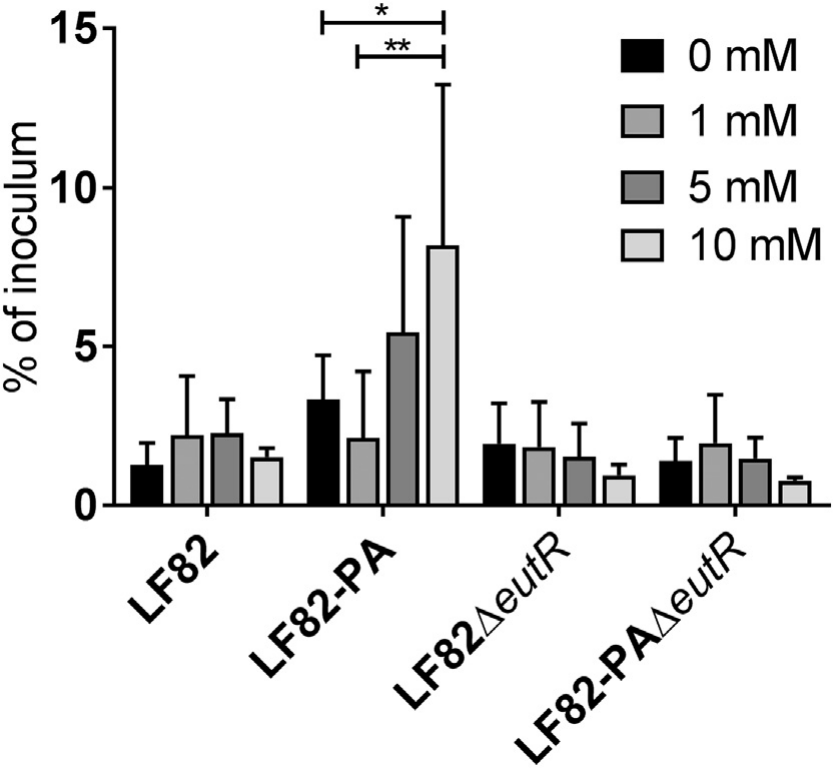
Extracellular ethanolamine increases intracellular replication of LF82-PA. Intra-macrophage (RAW264.7) survival and replication of wild type, PA-adapted, and LF82ΔeutR at 24 h post-infection with or without ethanolamine supplementation. For all values, the mean ± SD of three independent biological replicates are shown. Statistical analyses were preformed using GraphPad Prism, with data analysed by two-way ANOVA (p < .05 *; *p* < .01 **; p < .001 ***).

**Supplementary Fig. S2:**
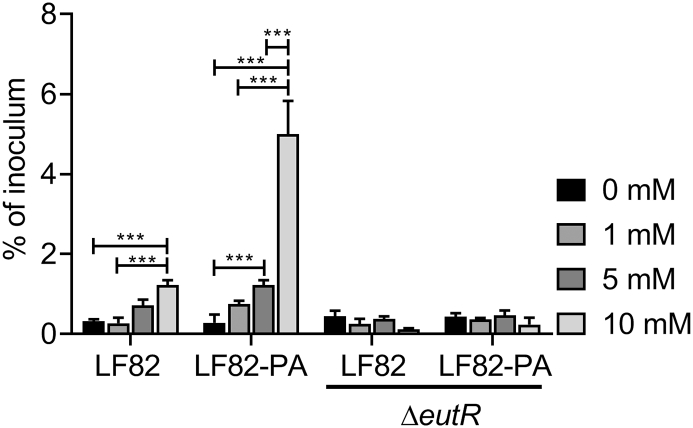
Extracellular ethanolamine increases intracellular replication of LF82-PA in THP-1 cells. Intra-macrophage (THP-1) survival and replication of wild type, PA adapted, and Δ*eutR* mutants of each at 4 h post-infection with or without ethanolamine supplementation. For all values, the mean ± SD of three independent biological replicates are shown. Statistical analyses were preformed using GraphPad Prism, with data analysed by two-way ANOVA (*p* < .05 *; *p* < .01 **; *p* < .001 ***).

Rapid intracellular replication in macrophages is a key phenotypic trait in AIEC [1,3,51]. However the mechanism behind this increased replication has remained elusive. In order to examine the effect of human intestinal levels of extracellular ethanolamine on the intra-macrophage replication of LF82, we infected RAW264.7 macrophages with LF82 and LF82 that had been exposed to PA (LF82-PA) [22,31]. Supplementation of ethanolamine did not affect replication of wild type LF82 after 24 h at any concentration (Fig. 3). However, replication of LF82-PA within macrophages significantly increased in response to ethanolamine in a dose dependent manner (Fig. 3). Ethanolamine addition did not affect replication of the LF82Δ*eutR* strain or LF82-PAΔ*eutR*, which are unable to metabolise ethanolamine due to the removal of the regulator of the ethanolamine utilization operon *eutR* (Fig. 3). While RAW264.7 cells are commonly used to study AIEC virulence, to ensure the observed effects were not specific to murine macrophages this was repeated within the human monocyte THP-1 cell line where again ethanolamine significantly increased intracellular replication in a dose-dependent and *eut* dependent fashion (supplementary Fig. S2). Collectively, these data indicate that the intestinal short chain fatty acid PA induces increased survival and replication of LF82 within macrophages in the presence of concentrations of ethanolamine found in the human intestine.

### 3.4 The PA-driven enhanced intracellular replication phenotype is mirrored in other clinical AIEC isolates

**Supplementary Fig. S3:**
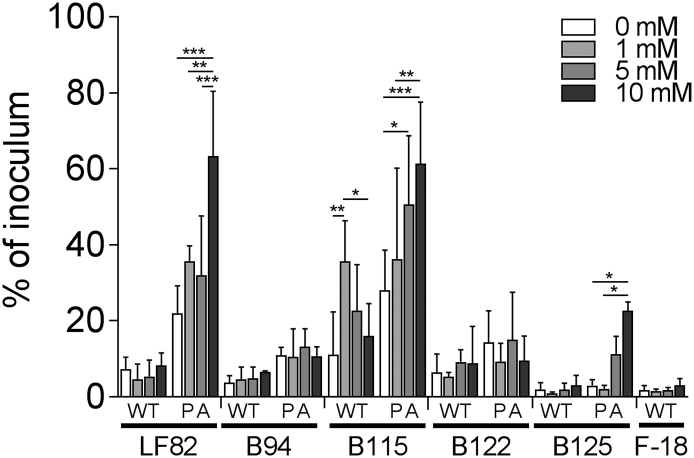
Extracellular ethanolamine increases intracellular replication of clinical *E. coli* isolates. Intra-macrophage (RAW 264.7) survival and replication of wild type and PA adapted LF82, clinical isolates B94, B115, B122 and B125 and commensal *E. coli*, F-18 24 h post-infection with or without ethanolamine supplementation. For all values, the mean ± SD of three independent biological replicates are shown. Statistical analyses were preformed using GraphPad Prism, with data analysed by two-way ANOVA (p < .05 *; p < .01 **; p < .001 ***).

*E. coli* isolated from intestinal biopsies of pediatric patients with active CD were examined for their ability to replicate intracellularly in macrophages in the presence of extracellular ethanolamine. The characteristics of these isolates has been determined previously with all isolates exhibiting an AIEC phenotype after PA-exposure with an ability to; adhere to and invade intestinal epithelial cells, replicate within macrophages and form biofilms both aerobically and anaerobically [29]. PA pre-exposure resulted in significantly increased intracellular replication in comparison to the unexposed wild type in two of four strains, whilst in the two others the increase was not significant (Supplementary Fig. S3). The increasing concentrations of ethanolamine significantly increased intracellular replication of isolates B115 and B125 in a dose dependent manner. Due to an inability to metabolise PA, we were unable to generate an adapted strain of the commensal *E. coli* F-18 strain. In any case, F-18 showed a distinct lack of intracellular replication. Collectively, these data indicate that the intestinal SCFA PA induces increased survival and replication of LF82 and clinically relevant *E. coli* within macrophages in the presence of physiologically relevant concentrations of ethanolamine.

### 3.5 Expression of the eut operon correlates with inflammatory status in pediatric CD patients

Given the significance of ethanolamine utilization in facilitating *in vitro* infection by LF82 we examined faecal samples from pediatric CD patients, before and after treatment, compared to healthy controls to determine any relevance of the *eut* operon to disease status. All CD patients had active disease at treatment initiation (wPCDAI >12.5). By the end of treatment with exclusive enteral nutrition (EEN), (the first-line therapy for pediatric CD), 8/10 (80%) of patients had clinically improved (wPCDAI decrease >17.5), with 6/10 (60%) of patients having entered clinical remission (mean wPCDAI treatment start: 42.25 [23.4]; wPCDAI treatment end: 11 [12.4]). There was also a significant decrease in faecal calprotectin levels during the course of treatment from initial mean values of 1561 mg/Kg (SD:596) at the start of treatment, decreasing to 1037 mg/Kg (592, 1614) by the end of EEN (Supplementary Table S3).

**Fig. 4:**
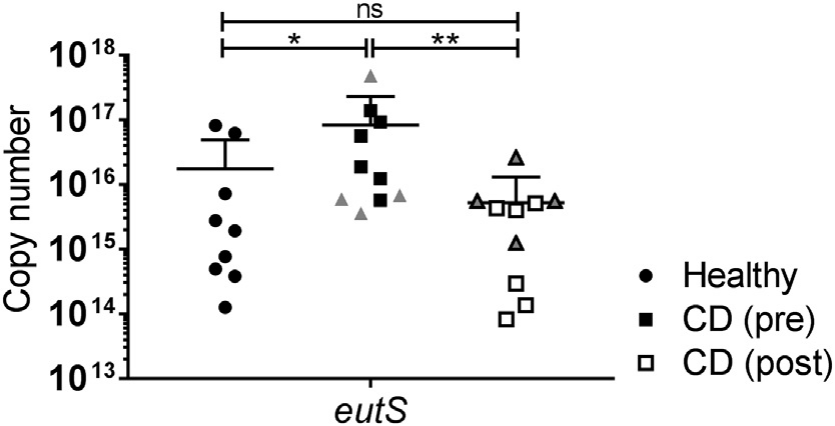
qRT-PCR *of eutS* in healthy patients, active Crohn's disease patients and Crohn's disease patients following EEN*.* Ethanolamine utilization was determined by abundance of *eutS* transcripts. *eutS* was amplified using primers designed against LF82. Transcript levels were normalized to 16S rRNA transcripts. Healthy samples (*n* = 9); Crohn's disease samples pre-treatment (CD pre; *n* = 10); Crohn's disease samples post-treatment (CD post; n = 10). Crohn's disease samples pre- and post-treatment were paired. Patients were marked as responders (squares) and non-responders (triangles) based on their drop in calprotectin levels (Supplementary Table S1). Statistical analyses were preformed using GraphPad Prism, with data analysed by one-way ANOVA followed by Dunns multiple comparisons post-test (p < .05 *; p < .01 **).

**Supplementary Fig. S4:**
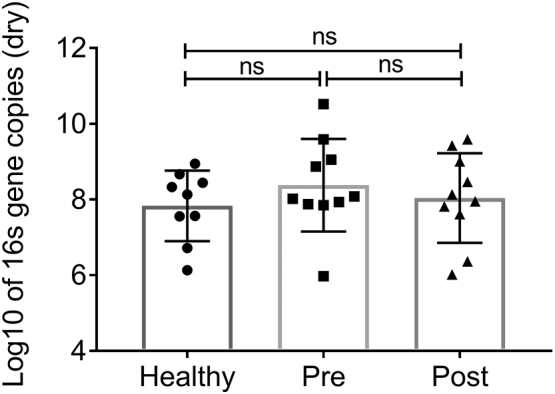
Total *E. coli* was determined by abundance of *16S* transcripts amplified using primers designed against *E. coli*. Healthy samples (*n* = 9); Crohn's disease samples pre-treatment (CD pre; *n* = 10); Crohn's disease samples post-treatment (CD post; n = 10). Crohn's disease samples pre- and post-treatment were paired. Statistical analyses were preformed using GraphPad Prism, with data analysed by one-way ANOVA followed by Dunns multiple comparisons post test (p < .05 *; p < .01 **).

Quantitative RT-PCR (qRT-PCR) indicated an increase in *E. coli eutS* expression in children with active CD compared to healthy controls (Fig. 4; Fold change increase: >4.72; *P* < .02). A significant decrease in *eutS* expression was observed post-EEN treatment across all CD patients (Pre *vs* Post Fig. 4; Fold change decrease: >15.64; *P* < .01). This drop in *eutS* copy number was most significant in patients with reduction in their faecal calprotectin levels as an indicator of colonic inflammation (Supplementary Table S3). Subsequent analysis of total *E. coli* numbers using 16S gene copy number revealed no significant differences between healthy controls, CD patients pre-treatment and the same patients post-treatment (Supplementary Fig. S4). This indicates that the observed differences in *eutS* copy number are not due to fluctuating *E. coli* levels but due to transcriptional differences.

## 4 Discussion

The role of AIEC in the pathology of CD has remained an enigma since the recovery of the first strain from a CD patient nearly 20 years ago [52]. Subsequent work indicating their predominance in both CD and colorectal cancer patients has led to numerous studies looking for a common denominator linking all distantly related AIEC strains that are now found widely spread across all *E. coli* subtypes [53,54]. Here we present evidence indicating a common intestinal carbon and nitrogen source, ethanolamine, can be readily used by the AIEC type strain LF82 and other AIEC strains isolated from CD patients. The significance of ethanolamine in AIEC virulence however is three-fold; its utilization is stimulated by the intestinal short chain fatty acid PA; it is available to AIEC enabling increased growth within macrophages when added extracellularly at intestinal concentrations, and its utilization within the intestine directly correlates with inflammatory status of pediatric CD patients.

Our previous work indicated that prior-exposure of AIEC to PA results in a pathogen with enhanced abilities to adhere to and invade intestinal epithelial cells, tolerate acidic conditions, form better biofilms and persist in an animal model of infection [29]. Here we show that PA pre-exposure also acts as a metabolic signal, stimulating degradation of ethanolamine. The action of PA as a positive signal for colonization by AIEC of the inflamed intestine is in stark contrast to that of pathogens such as *Campylobacter* spp. and *S.* Typhimurium, where the high PA concentration of the caecum and colon have been shown to represses virulence and colonization [[33], [34], [35], [36], [37], [38]]. This ability to withstand the toxic effects of PA and use it as a positive inducer of virulence sets AIEC and a small number of other pathogens such as *Mycobacterium tuberculosis*/*avium* apart [[55], [56], [57]].

Notably, after exposure to PA the LF82-PA strain was more readily able to utilize ethanolamine as a carbon source, resulting in an increased rate of growth and increased biomass (Fig. 1). This observation was not surprising, given that growth on PA resulted in expression of *eut* (Fig. 2f) but it also enabled LF82-PA to significantly increase intra-macrophage replication (Fig. 3). Rapid intra-macrophage growth is a recognized feature of the AIEC pathotype [1,3,51] and may play a critical role in facilitating persistence in the intestine given numbers of macrophages and dendritic cells are increased in the mucosa of CD patients [58]. As ethanolamine is also more readily available for consumption under these conditions and our work demonstrates that increased extracellular ethanolamine is available to AIEC, ethanolamine may be a crucial carbon source in facilitating AIEC persistence in immune cells in the intestine [21]. Similarly, increased intra-macrophage proliferation due to the presence of extracellular ethanolamine has been demonstrated previously in *S*. Typhimurium indicating additional persistence strategies such as inhibition of programmed cell death may be needed to facilitate long-term intracellular ethanolamine use [25,59]. The driver for predominance of AIEC in CD, as opposed to other intestinal enterobacterial pathogens such as *S.* Typhimurium and *Y. enterocolitica*, remains unknown. However the ability of AIEC to positively respond to the normally antimicrobial SCFA PA may underlie this unique ability. While other intestinal pathogens are directly inhibited by this potent antimicrobial, AIEC can utilize PA, incorporate it, and respond to its presence [29,[33], [34], [35], [36], [37], [38]]. Therefore, in the presence of PA, upregulation of the *eut* operon may give AIEC a distinct competitive advantage in the inflamed, ethanolamine replete CD intestine. Whether this capability is conserved across a wide range of *E. coli* strains or is distinct to AIEC is not yet known, but if widely conserved it may explain why *E. coli* as opposed to other bacteria dominate the CD microbiome. Indeed, utilization of ethanolamine may benefit AIEC through induction of a positive feedback loop whereby ethanolamine use leads to AIEC proliferation resulting in further inflammation and ethanolamine release.

We observed the secretion of MCPs inside OMVs during growth on PA (Fig. 2b and c). The suggested association between AIEC and the 1,2-PD utilization operon (*pdu*) and the close metabolic relationship between 1,2-PD degradation and PA production lead us to hypothesize that upregulation of the *pdu* operon would be responsible for their production [16,17,60]. Surprisingly, our analysis indicated these PA-induced MCPs were encoded by the *eut* operon which allows for degradation of ethanolamine (Fig. 2f). Ethanolamine confers on a number of pathogens an important metabolic advantage in out-competing the host microbiota during episodes of inflammation [[21], [22], [23], [24]]. The *eut* derived MCP is known to enhance *E. coli* and *S. enterica* proliferation in diverse environments, including on food products, in *Caenorhabditis elegans* models of infection, during growth on bovine intestinal content, and in a murine model of infection [21,22,24,27]. It is therefore plausible that PA exposure provides AIEC with an enhanced ability to survive within regions of the intestinal tract that other pathogens cannot, signaling for upregulation of the genes necessary for ethanolamine metabolism.

As our *in vitro* findings indicated that *eut* metabolism conferred a significant growth advantage on AIEC in the context of infection, we next sought to investigate the significance of this in the context of AIEC predominance in CD patients. Using faecal samples taken over an eight week period we determined the levels of *eut* expression in patients both before and after induction treatment and compared these to healthy controls. All CD patients had undertaken an exclusive enteral nutrition (EEN) diet for the eight-week period after the initial sample was taken and faecal calprotectin levels were used to assess changes in colonic inflammation and to assign patients to either responder or non-responder groups (Supplementary Table S3). *eutS* levels were increased by >4.7 fold in CD patients compared to healthy controls indicating a significant increase in ethanolamine use in these patients (Fig. 4). However this effect was reversed by EEN treatment with both inflammation and *eutS* levels significantly reduced (>15.6 fold) and *eutS* levels not being significantly different post-treatment to healthy controls. While the availability of ethanolamine during inflammation is not surprising given previous reports, the direct correlation between CD activity and *E. coli eutS* transcript levels was unexpected. Samples from those patients who responded best to the treatment were shown to have the most significant reduction in *eutS* levels, further indicating a correlation between ethanolamine use and *E. coli* in the CD intestine. This is possibly explained by the observed drop in intestinal propionate levels seen after EEN treatment [45]. This reduction in the signaling molecule for AIEC metabolism of ethanolamine would lead to the type of *eut* transcription drop observed here. Further validation of this in another dataset of patients that have undergone a different mode of treatment induction would be a useful extension of this work.

This work highlights a new AIEC phenotypic trait that exhibits a direct correlation with severity of CD. Monitoring of *eut* expression within the CD intestine shows potential as a useful biomarker for monitoring severity of CD and overgrowth of *E. coli* in CD, perhaps predicting useful interventions using direct targeting of *E. coli* to alleviate disease [45]. This work also raises the possibility that other pathogens associated with CD may utilize similar metabolic strategies.

The following are the supplementary data related to this article.

Supplementary material Image 1

## Funding sources

This work was funded by Biotechnology and Biological Sciences Research Council (BBSRC) grants BB/K008005/1 & BB/P003281/1 to DMW; by a Tenovus Scotland grant to MJO; by Glasgow Children's Hospital Charity, Nestle Health Sciences, Engineering and Physical Sciences Research Council (EPSRC) and Catherine McEwan Foundation grants awarded to KG; by a Natural Environment Research Council (NERC) fellowship (NE/L011956/1) to UZI; The IBD team at the Royal Hospital for Children, Glasgow are supported by the Catherine McEwan Foundation and Yorkhill IBD fund. RKR and RH are supported by NHS Research Scotland Senior fellowship awards. The funders had no role in study design, data collection, data analysis, interpretation, writing of the report.

## Declarations of interest

Mr. Logan's PhD studentship was funded, in part, from the EPSRC and Nestle Health Science, during the conduct of the study. Dr. Hansen reports consultancy fees and conference support from Nutricia and consultancy fees from 4D Pharma. Dr. Gerasimidis reports grants from Nestle Global, during the conduct of the study; grants and personal fees from Nutricia, outside the submitted work. Dr. Russell reports speaker's fees, travel support and medical board meeting participation from Abbvie, Therakos, Celltrion, Nestle and Ferring Pharmaceuticals, outside the submitted work. No conflicts of interest, financial or otherwise, are declared by the other authors.

## Author contributions

MJO developed the initial concept, designed and performed the experiments, analysed the data and prepared the manuscript; ML recruited participants, collected and curated samples; SAJ helped develop the initial concept, assisted in experimental design and critically appraised the manuscript; AM provided technical assistance throughout the study; GF conducted intra-macrophage replication experiments; GLH and RH supplied clinical isolates and patient specific details from the BISCUIT study; RP and EP extracted DNA from faecal samples and conducted qPCR; RKR, ML and RH recruited participants and followed them up; KG & RKR designed the clinical study, applied for funding and ethical approvals; RKR, UZI, KG supervised and co-ordinated the clinical study; DMW developed the initial concept, designed the experiments, analysed the data and prepared the manuscript. All authors contributed to editing the manuscript for publication.

## General

We thank Professor José R. Penades for his constructive criticism of the manuscript.
