# Supplementary material for: Inflammation associated ethanolamine facilitates infection by Crohn's disease-linked adherent-invasive Escherichia coli
Source: eBioMedicine. 2019 Apr 26;43:325–32. doi: 10.1016/j.ebiom.2019.03.071 (PMC6557746; doi:10.1016/j.ebiom.2019.03.071)
Supplement: Supplementary file 1 — Supplementary material [file mmc1.pdf]

Supplementary Table S1 Strain information

Strain	Description	Reference
LF82	AIEC wild type strain	Prof. Daniel Walker, University of Glasgow
LF82-PA	LF82 pre-exposed to 20 mM propionic acid	*Ormsby et al., 2018*
LF82Δ*eutR*	LF82 with *eutR* knocked out	*This study*
LF82-PAΔ*eutR*	LF82-PA with eutR knocked out	*This study*

Supplementary Table S2 List of primers used in this study

Primer	Sequence 5' - 3'
eutR KO For	ATTAACCTGTCGGAGGTGCCGGGTGTCCTACAACACCCGGCATTAACATCGTGTAGGCTGGAGCTGCTTC
eutR KO Rev	GAAGCGGCTTTAAGTGAGTTTATTAAGGTCAGGGATTGGGTGTAACTCCCATGGGAATTAGCCATGGTCC
Δ*eutR* Check For	CAACCCGTAAAGGAACCCGA
Δ*eutR* Check Rev	TTTATGCAGGCGGTAGGCAA
16s For	TGAGAATGTGCCTTCGGGAG
16s Rev	TTGCGGGACTTAACCCAACA
pduA For	AGGCTTAACTGCCGCCATAG
pduA Rev	CACCAGCCCGGAACCTATTT
prpB For	TATTCGCCGTATCACCGACG
prpB Rev	CGCCACGTTAAAGGCTGAAG

Supplementary Table S3 Patient and clinical response information

Sample ID	Sample	Post treatment	Gender	Age (years)	wPCDAI (Pre)	Calprotectin (Wet -Pre)	wPCDAI	Calprotectin (Wet -Post)	wPCDAI decrease	<12.5 wPCDAI	Calprotectin decrease
108A	Healthy	-	Male	11.0	3.7	-	-	-	-	-	-
124A	Healthy	-	Female	7.4	14.5	-	-	-	-	-	-
127A	Healthy	-	Male	7.4	8.4	-	-	-	-	-	-
128A	Healthy	-	Male	5.1	94.9	-	-	-	-	-	-
129A	Healthy	-	Female	13.7	15.2	-	-	-	-	-	-
130A	Healthy	-	Male	12.1	3.1	-	-	-	-	-	-
133A	Healthy	-	Male	7.0	12.1	-	-	-	-	-	-
134A	Healthy	-	Male	4.5	156.4	-	-	-	-	-	-
135A	Healthy	-	Male	9.6	100.1	-	-	-	-	-	-
012A	Crohn's Disease	Responder	Female	12.4	22.5	1813.0	0.0	1023.5	22.5	Y	789.5
018A	Crohn's Disease	Responder	Male	14.1	17.5	1781.9	5.0	2.8	12.5	Y	1779.1
023A	Crohn's Disease	Responder	Male	10.3	52.5	1744.1	0.0	1207.4	52.5	Y	536.8
201A	Crohn's Disease	Responder	Female	8.4	95	2440.1	17.5	879.1	77.5	N	1561.0
206A	Crohn's Disease	Responder	Female	8.5	27.5	1612.5	0.0	1110.0	27.5	Y	502.5
217A	Crohn's Disease	Responder	Male	8.7	60	706.2	0.0	256.8	60	Y	449.4
005A	Crohn's Disease	Non-Responder	Male	16.2	37.5	1699.0	32.5	1791.5	5	N	-92.5
009A	Crohn's Disease	Non-Responder	Female	8.2	52.5	1774.1	27.5	1809.6	25	N	-35.5
203A	Crohn's Disease	Non-Responder	Male	15.8	32.5	1683.6	20.0	1554.5	12.5	N	129.1
216A	Crohn's Disease	Non-Responder	Female	15.1	25	350.6	7.5	703.7	17.5	Y	-353.1

Supplementary Table S4 **Resource table**

REAGENT or RESOURCE	SOURCE	IDENTIFIER
**Antibodies**		
n/a		
**Bacterial and Virus Strains**		
Escherichia coli	Prof. Daniel Walker, University of Glasgow	LF82: AIEC wild type strain
Escherichia coli	*Ormsby et al., 2018*	LF82-PA: LF82 pre-exposed to 20 mM propionic acid
Escherichia coli	*This study*	LF82ΔeutR: LF82 with *eutR* knocked out
Escherichia coli	*This study*	LF82-PAΔeutR: LF82-PA with eutR knocked out
**Biological Samples**		
Human Faecal samples	Paediatric gastroenterology clinics at the Royal Hospital for Children, Glasgow	www.clinicaltrials.gov (NCT02341248)
**Chemicals, Peptides, and Recombinant Proteins**		
Lysogeny Broth (LB)	LabM a Neogen company	NCM0088A
Bacto Agar	Formedium	AGA02
Disodium hydrogen phosphate dihydrate	Merck	10028-24-7
Sodium chloride	Merck	7647-14-5
Ammonium chloride	Fisher	12125-02-9
Potassium hydrogen phosphate	Merck	.7758-11-4
Trace metal solution	http://cshprotocols.cshlp.org/content/2006/1/pdb.rec630.full?text_only=true	Cold Sping harbour protocols
Magnesium sulphate	VWR Chemicals	7487-88-9
Calcium chloride	Fisher Scientific	22189-08-8
Thiamine hydrochloride	Fisher Scientific	67-03-8
Iron chloride	Fisher Scientific	10025-77-1
Ethylenediaminetetraacetic Acid (EDTA)	Fisher Scientific	60-00-4
Taurocholic acid	Fisher Scientific	345909-26-4
D-glucose	Sigma	50-99-7
Sodium propionate	Sigma	137-40-6
1,2-Propanediol	Sigma	57-55-6
Ethanolamine	Sigma	141-43-5
RPMI-1640	Thermofisher	31870025
Foetal calf serum	Fisher Scientific	11573397

Supplementary Table S4 **Resource table**

REAGENT or RESOURCE	SOURCE	IDENTIFIER
L-glutamine	Fisher Scientific	15430614
No-Carbon-E (NCE media)	Cheng *et al*., 2011	-
Cyano-cobalamin	Fisher Scientific	68-19-9
Ammonium molybdate	Fisher Scientific	12054-85-2
RNAprotect	Qiagen	76526
RNAlater	Thermofisher	10391085
polymeric casein-based liquid feed	Modulen; Nestlé UK Ltd	140447
Penicillin/streptomycin	Sigma	P4333
Acetonitrile	Sigma	75-05-8
Formic acid	Sigma	64-18-6
Dimethyl Sulfoxide (DMSO)	Sigma	67-68-5
**Critical Commercial Assays**		
Calpro ELISA kit (PhiCal)`	Calpro	9053
RNeasy PowerMicrobiome Kit	Qiagen	26000-50
Affinity Script cDNA multi-temp Synthesis Kit	Agilent	200436
PerfeCTa SYBR Green FastMix	Quanta Biosciences - VWR	733-1382
Anaerobic condition generator	Ocoid AnaeroGen	AN0025
ExoBacteria OMV Isolation Kit	System Biosciences, California, USA	EXOBAC100A-1
**Deposited Data**		
**Experimental Models: Cell Lines**		
RAW264.7 murine macrophage cell line	American Type Culture Collection (ATCC)	RAW 264.7 (ATCC® TIB71™)
THP-1 Human monocytic leukaemia cells	European Collection of Authenticated Cell Cultures (ECACC)	THP 1 (ECACC 88081201)
**Experimental Models: Organisms/Strains**		

Supplementary Table S4 **Resource table**

REAGENT or RESOURCE	SOURCE	IDENTIFIER
**Oligonucleotides**		
eutR KO For	ATTAACCTGTCGGAGGTGCCGGGTGTCCTACAACACCCGGCATTAACATCGTGTAGGCTGGAGCTGCTTC	Sigma
eutR KO Rev	GAAGCGGCTTTAAGTGAGTTTATTAAGGTCAGGGATTGGGTGTAACTCCCATGGGAATTAGCCATGGTCC	Sigma
Δ*eutR* Check For	CAACCCGTAAAGGAACCCGA	Sigma
Δ*eutR* Check Rev	TTTATGCAGGCGGTAGGCAA	Sigma
16s For	TGAGAATGTGCCTTCGGGAG	Sigma
16s Rev	TTGCGGGACTTAACCCAACA	Sigma
pduA For	AGGCTTAACTGCCGCCATAG	Sigma
pduA Rev	CACCAGCCCGGAACCTATTT	Sigma
prpB For	TATTCGCCGTATCACCGACG	Sigma
prpB Rev	CGCCACGTTAAAGGCTGAAG	Sigma
**Recombinant DNA**		
**Software and Algorithms**		
GraphPad Prism	v7.0c, Prism	-
		-
**Other**		
